# Wnt signaling pathways in myocardial infarction and the therapeutic effects of Wnt pathway inhibitors

**DOI:** 10.1038/s41401-018-0060-4

**Published:** 2018-07-12

**Authors:** Wen-bin Fu, Wei Eric Wang, Chun-yu Zeng

**Affiliations:** Department of Cardiology, Daping Hospital, Third Military Medical University, Chongqing, 400042 China

**Keywords:** Wnt pathway inhibitors, myocardial infarction, cardiac repairing, therapeutic effect

## Abstract

Myocardial infarction (MI) is one of the most serious health threats, resulting in huge physical and economic burdens worldwide. Wnt signaling pathways play an important role in developmental processes such as tissue patterning, cell differentiation and cell division. Appropriate regulation of the activities of Wnt signaling pathways is also important for heart development and healing in post-MI heart. Moreover, Wnt pathway inhibitors have been identified as novel antitumor drugs and applied in ongoing clinical trials. This research progress has generated increasing interests for investigating the effects of Wnt pathway inhibitors on MI healing. In this short review, we summarize the roles of Wnt signaling pathways in post-MI heart and the therapeutic effects of Wnt pathway inhibitors on MI, and discuss the underlying mechanisms of Wnt pathway inhibitors in cardiac repairing.

## Introduction

Myocardial infarction (MI) is one of the leading causes of morbidity and mortality and threatens human health worldwide [1]. In spite of the therapeutic function of drugs such as adrenoceptor blockers, calcium antagonists and renin-angiotensin system inhibitors [2], the progression of MI and pathological remodeling are still irreversible. Recent studies agree on the fact that adult mammalian cardiomyocytes are able to regenerate but with a very limited efficacy [3]. This fact evokes further studies to discover novel drug candidates that aim to reduce pathological remodeling and stimulate cardiac regeneration.

Wnt signaling pathways are essential in heart development and active in post-MI adult heart [4, 5]. Recent breakthroughs in the studies on the Wnt signaling pathways have revealed new points of intervention that may lead to novel drug targets for small molecular weight compounds. Wnt pathway inhibitors are primarily designed for the treatment of tumors and have been approved in clinical trials [6, 7]. The effect of Wnt pathway inhibitors in the field of cardiac protection has aroused sustained attention towards applying these inhibitors to treat MI [8–10]. In this review, we summarize the regulating mechanisms of Wnt signaling pathways in MI and the effects of Wnt pathway inhibitors against MI.

## Wnt signaling pathways and cardiomyogenesis

**Fig. 1:**
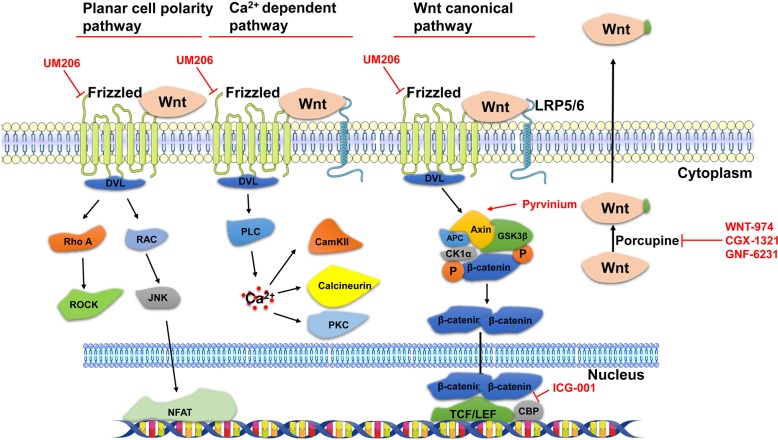
Wnt signaling pathways and the intervention targets of Wnt pathway inhibitors. The secretion of Wnt proteins is dependent on palmitoylation by Porcupine. Frizzled and low-density lipoprotein receptor (LRP) proteins are membrane receptors for binding Wnt proteins. In the canonical Wnt pathway, Wnt binding leads to β-catenin nuclear translocation (after dissociation from a complex with Axin, glycogen synthase kinase 3β (GSK3β), adenomatous polyposis coli (APC) and casein kinase 1α (CK1α)), causing its interaction with TCF/LEF transcription factors and gene transcription. In the Wnt/PCP pathway, Wnt binding results in RhoA/ROCK and Rac/Jnk/NFAT pathway transduction. In the Wnt/Ca^2+^ pathway, Wnt binding leads to phospholipase C (PLC) activation and the accumulation of intracellular Ca^2+^, which then leads to the activation of calmodulin-dependent kinase II (CamKII), calcineurin and protein kinase C (PKC). UM206 targets the Frizzled receptor family; pyrvinium inhibits Axin degradation; ICG-001 inhibits the interaction between β-catenin and CBP; and WNT-974, CGX-1321 and GNF-6231 are porcupine inhibitors

Wnt was first discovered as *Int-1*, and then it was found to be a homologous gene of the *wingless* gene in Drosophila [11]. Wnt signaling pathways include the canonical Wnt/*β-catenin* pathway, non-canonical Wnt/planar cell polarity (PCP) pathway and Wnt/Ca^2+^ dependent pathway (Fig. 1) [6]. All three pathways are activated by the binding of a Wnt-protein ligand to a Frizzled family receptor, which conducts signal to the Dishevelled *(Dvl)* protein inside the cell [12]. The secretion of Wnt proteins is dependent on palmitoylation by Porcupine [13]. Another docking protein family named low-density lipoprotein receptor (*LRP*) is also found in the Wnt/Frizzled complex [14]. In the canonical Wnt pathway, *β-catenin* is phosphorylated and degraded by a destruction complex, including *Axin*, glycogen synthase kinase 3β (*GSK3β*), adenomatous polyposis coli (*APC*), and casein kinase 1α (*CK1α*). Wnt stimulation phosphorylates *Dvl* protein, dissociates *β-catenin* and induces the nuclear translocation of *β-catenin*, leading to its interaction with *TCF/LEF* transcription factors and gene transcription [15]. In the Wnt/PCP pathway, *Dvl* phosphorylation results in *RhoA/ROCK* and *Rac/Jnk/NFAT* pathway activation [16]. In the Wnt/Ca^2+^ pathway, phospholipase C (*PLC*) is activated and induces the accumulation of intracellular Ca^2+^, leading to the activation of calmodulin-dependent kinase II (CamKII), calcineurin and protein kinase C (PKC) [17].

The mammalian heart is developed from the lateral plate mesoderm, which fuses together to form the heart tube and subsequently organizes the ventricle and atria [18]. Wnt signaling pathways exert bi-directional regulatory effects at different stages of cardiomyogenesis. The Wnt signaling pathway is activated during the formation of the early embryo to the lateral plate mesoderm but is subsequently inhibited to ensure heart development into its proper size [4]. Wnt activation at the gastrulation stage is essential for the maintenance of undifferentiated progenitor cells [19, 20], whereas Wnt inhibition at the post-gastrula stages promotes cardiomyocyte differentiation [21, 22]. Different Wnt ligands participate in progress of mesoderm induction and later cardiomyocyte differentiation. *Wnt-3* and *Wnt-8a* regulate mesoderm induction through canonical signaling, whereas *Wnt-2*, *Wnt-5a/b*, and *Wnt-11* regulate cardiomyocyte differentiation via non-canonical signaling [23]. Dissociative *β-catenin* is the central player of the canonical Wnt pathway, and its nuclear accumulation is a hallmark of Wnt signaling activation [6]. The Wnt/β-catenin signaling pathway is also crucial in the formation of second heart field derivatives, such as cardiac outflow tract and right ventricle [24, 25]. Moreover, the inhibition of *β-catenin*-mediated transcription is linked with atrial and ventricular growth in zebrafish, indicating its role in cardiomyocyte proliferation [26].

## Wnt signaling pathways and MI in mammalian heart

### Activation of Wnt signaling pathways after MI

The pathological process of MI injury includes three major phases: inflammatory reaction, the formation of granulation tissue and fibrosis [27]. The infiltration of inflammatory cells leads to the release of chemokines and cytokines and the recruitment of macrophages. Then, necrotic debris is cleared, and angiogenesis is activated. Finally, myofibroblasts generate collagen to replace the lost cardiomyocytes and provide compensatory contraction function [28, 29].

There is increasing evidence showing that Wnt signaling is triggered during the pathological process of MI injury. Quantitative analysis for the expression of Wnt proteins has shown strong *Wnt-2*, *Wnt-4*, *Wnt-10b*, and *Wnt-11* upregulation 5 days after MI [30]. *Wnt-1* was upregulated from 1 to 14 days after MI, and upregulated *Wnt-4* was observed from 7 to 14 days after MI in a subsequent study [31]. *Axin2* and *LEF1* are both Wnt target genes and serve as readouts of Wnt signaling intensity. Using a fate-mapping technique with the *Axin2* promoter and LacZ labeling, Wnt signaling is proved to be activated in cardiomyocytes located at infarct border zone [32]. The intensity of Wnt signaling peaks at 7 days after MI and then is gradually attenuated in fibroblasts, endothelial cells, and progenitor cells [32]. Using TopGAL mouse, which expresses the marker β-gal under the control of *TCF/LEF1*, increased Wnt signaling activity has been detected 4 days after MI [30]. According to the above results, sustained Wnt activation is associated with the pathological phases after MI, including inflammation, angiogenesis and fibrosis.

### Wnt signaling pathways and inflammation in MI

The inflammatory reaction is first activated in response to MI, which aims at removing necrotic debris and healing the infarct but also leads to cardiac remodeling and dysfunction [33]. It has been reported that a subtype of Wnt proteins, *Wnt-5a*, which is exclusively expressed in cardiomyocytes [8], promotes the release of *IL-1*, *IL-6* and *IL-8* from mononuclear cells, indicating the pro-inflammatory effect of Wnt signaling [34]. Moreover, SFRPs, which are endogenous Wnt pathway inhibitors, can protect MI injury by modulating the inflammatory response. Better scar formation and cardiac hemodynamic parameters have been demonstrated when bone marrow cells (BMCs) with overexpressed *SFRP1* are transplanted into the infarcted border zone by blocking leukocyte activation and cytokine production [35]. Similarly, *SFRP5* is able to inhibit inflammatory cytokine and chemokine gene expression in ischemic heart [36]. Furthermore, the deletion of Wnt inhibitory factor 1 (WIF1*)* leads to more inflammatory monocytes and severe adverse remodeling, whereas cardiomyocyte-specific *WIF1* overexpression attenuates monocyte response and improves cardiac function [37]. Increasing evidence has revealed that non-canonical Wnt signaling pathways are most likely involved in inflammatory processes in ischemic heart. Inflammatory cell differentiation and pro-inflammatory cytokine release can be triggered by the non-canonical Wnt signaling pathway via the *CamKII /NFAT* pathway [38]. In addition, *SFRP5* functions to mitigate inflammation through the non-canonical *Wnt-5a/JNK* signaling pathway [36].

### Wnt signaling pathways and angiogenesis in MI

Angiogenesis is reflected as newly formed vessels by endothelial cells, which contribute to cardiac repair and functional recovery after MI. A previous study showed that the Wnt signaling pathway was activated in endothelial cells of the infarct area, which were identified by the accumulation of *β-catenin* [39]. Conditional overexpression of *β-catenin* in endothelial cells shows progressive cardiac dysfunction via *ErbB* signaling, indicating Wnt inhibition as a therapeutic strategy for heart failure [40]. Indeed, many kinds of negative Wnt regulators have demonstrated a pro-angiogenesis effect in post-MI heart. Genetic overexpression of *SFRP1* can increase capillary density in the scar of MI by inhibiting the accumulation of cytosolic *β-catenin* [41]. Dickkopf2 (DKK2), known as an another Wnt inhibitor, can stimulate the angiogenic sprouting of endothelial cells after MI via LRP6/APC activation, but Dickkopf1 (*DKK1*) has shown a diminished role in regulating angiogenesis [42]. However, it has been reported that NP12, which can stabilize *β-catenin* via inhibiting *GSK3β*, is able to promote angiogenesis and improve ventricular function following MI by activating the Wnt signaling pathway [43]. Moreover, Wnt-1-inducible secreted protein-1 (*WISP-1*), which is a responsive gene of Wnt activation [44], can promote angiogenesis in post-MI heart via regulating histone deacetylase [45].

### Wnt signaling pathways and fibrosis in MI

Cardiac fibrosis is a common process in remodeling hearts after MI that is formed by myofibroblast invasion and collagen secretion [28]. The canonical Wnt/*β-catenin* signaling pathway plays a dominant role in the regulation of cardiac fibrosis following MI. There is evidence showing that the down-regulation of *β-catenin* by aldehyde dehydrogenase-2 (ALDH2) activity leads to reduced cardiac fibrosis, which may be mediated by phosphorylated *GSK3β*, *Wnt-1*, and *WISP-1* [46]. In the meantime, the transfection of miR-154 inhibitors can also decrease the expression of *β-catenin* and myofibroblast proliferation via directly binding with *DKK2* [47]. Furthermore, TGFβ signaling plays a key role in the differentiation of myofibroblasts and interacts with the Wnt signaling pathway [48]. *Wnt-3a* can promote myofibroblast differentiation and *TGFβ* expression by triggering the canonical Wnt signaling pathway [49]. The Wnt signaling pathway can promote the release of *TGFβ* in the *β-catenin*-dependent pathway using a mouse model of autoimmune myocarditis [50], and loss of *β-catenin* function in cardiac fibroblasts results in improved cardiac function and suppressed interstitial fibroblasts in a mouse model of pressure overload [51]. However, in transgenic mice with specific *β-catenin* interruption in cardiac fibroblasts, impaired wound healing and decreased cardiac performance have been observed [31]. Conversely, the interruption of *β-catenin* in epicardial cells leads to compromised cardiac function after MI [31]. These discrepant observations may be explained by the complex roles of Wnt signaling pathways in different stages of MI and interactions with other pathways.

## The therapeutic effect of small molecule Wnt pathway inhibitors against MI in animal models

Pyrvinium is a drug approved by the FDA and identified as a Wnt inhibitor by inhibiting *Axin* degradation and stabilizing *β-catenin*, which can increase *Ki67*^+^ cardiomyocytes in the peri-infarct area and alleviate cardiac remodeling in a mouse model of MI [52]. UM206 is a peptide with a high homology to *Wnt-3a/5a*, and acts as an antagonist for Frizzled proteins to inhibit Wnt signaling pathway transduction. UM206 could reduce infarct size, increase the numbers of capillaries, decrease myofibroblasts in infarct area of post-MI heart, and ultimately suppress the development of heart failure [53]. ICG-001, which specifically inhibits the interaction between *β-catenin* and *CBP* in the Wnt canonical signaling pathway, can promote the differentiation of epicardial progenitors, thereby contributing to myocardial regeneration and improving cardiac function in a rat model of MI [54]. Small molecules invaliding Porcupine have been further studied, such as WNT-974, GNF-6231 and CGX-1321. WNT-974 decreases fibrosis in post-MI heart, with a mechanism of preventing collagen production in cardiomyocytes by blocking secretion of *Wnt-3*, a pro-fibrotic agonist, from cardiac fibroblasts and its signaling to cardiomyocytes [8]. The phosphorylation of *DVL* protein is decreased in both the canonical and non-canonical Wnt signaling pathways by WNT-974 administration [8]. GNF-6231 prevents adverse cardiac remodeling in a mouse model of MI by inhibiting the proliferation of interstitial cells, increasing the proliferation of *Sca1*^+^ cardiac progenitors and reducing the apoptosis of cardiomyocytes [9]. Similarly, we demonstrate that CGX-1321, which has also been applied in a phase I clinical trial to treat solid tumors (NCT02675946), inhibits both canonical and non-canonical Wnt signaling pathways in post-MI heart. CGX-1321 promotes cardiac function by reducing fibrosis and stimulating cardiomyocyte proliferation-mediated cardiac regeneration in a *Hippo/YAP*-independent manner [10]. These reports implicate that Wnt pathway inhibitors are a class of potential drugs for treating MI through complex mechanisms, including reducing cardiomyocyte death, increasing angiogenesis, suppressing fibrosis and stimulating cardiac regeneration.

## Conclusion

Both the canonical and non-canonical Wnt signaling pathways are essential for heart development and play an important role in adult heart with MI injury. The inhibition of Wnt signaling pathways has been demonstrated to be beneficial in MI via improving cardiac remodeling. These Wnt inhibitory compounds have been proven to be safe in clinical trials and can be potential drugs for treating MI. However, novel Wnt pathway inhibitors with minimized toxicity and a more exclusive effect on the heart are still needed.
